# Antihypertensive Effect of Ethanolic Extract from *Acanthopanax sessiliflorus* Fruits and Quality Control of Active Compounds

**DOI:** 10.1155/2018/5158243

**Published:** 2018-04-23

**Authors:** In Ho Jung, Sung Eun Kim, Yeong-Geun Lee, Dae Hyun Kim, Haneul Kim, Geum-Soog Kim, Nam-In Baek, Dae Young Lee

**Affiliations:** ^1^Department of Life and Nanopharmaceutical Science, College of Pharmacy, Kyung Hee University, Seoul 02447, Republic of Korea; ^2^Daehwa Pharmaceutical Co. Ltd., Seongnam 13488, Republic of Korea; ^3^Department of Oriental Medicine Biotechnology, Kyung Hee University, Yongin 17104, Republic of Korea; ^4^Department of Herbal Crop Research, National Institute of Horticultural and Herbal Science, RDA, Eumseong 27709, Republic of Korea

## Abstract

*Acanthopanax sessiliflorus* (Rupr. & Maxim.) Seem., which belongs to the Araliaceae family, mainly inhabits Korea, China, and Japan. Traditionally, *Acanthopanax* species have been used as treatment for several diseases such as diabetes, tumors, and rheumatoid arthritis. Especially, its fruits have many biological functions including antitumor, immunostimulating, antithrombosis, and antiplatelet activities. Recently, the extract of *A. sessiliflorus* fruit has been reported to have antithrombotic and antiplatelet activities related to the alleviation of hypertension. Therefore, we investigated the antihypertensive effect of ethanolic extract from *A. sessiliflorus* fruits (DHP1501) through *in vivo*, ex vivo, and *in vitro* studies. In this study, DHP1501 demonstrated free radical scavenging capacity, enhanced endothelial nitric oxide (NO) production, and inhibited angiotensin-converting enzyme (ACE) activity in spontaneously hypertensive rats (SHRs), resulting in the improvement of vascular relaxation and decrease in blood pressure in the hypertensive animal model. These results suggest that *A. sessiliflorus* fruit extract may be a promising functional material for the prevention and treatment of hypertension. Furthermore, this study demonstrated the utility of MS-based active compounds for the quality control of DHP1501.

## 1. Introduction

Hypertension, or the state of high blood pressure, is defined as above 140 mmHg systolic blood pressure (SBP) and 90 mmHg diastolic blood pressure (DBP). It is one of the most significant causes of mortality worldwide since elevated blood pressure is considered to be the leading risk factor for coronary artery disease and its complications such as heart failure, stroke, renal disease, and diabetes [1]. Hypertension is also regarded as the major risk factor for disability-adjusted life years worldwide according to the Global Burden of Disease study [1, 2]. The World Health Organization predicts that 1.5 billion people will suffer from hypertension by 2025 and that more than 7 million deaths yearly are likely to be caused by hypertension [3]. Owing to the global impact of hypertension, many studies have investigated antihypertensive medications and new therapeutic alternatives [4, 5]. There are various types of antihypertensive medications—such as angiotensin-converting enzyme (ACE) inhibitors, beta-blockers, and calcium channel blockers—owing to the many physiological mechanisms of blood pressure control including cardiac output, peripheral vascular resistance, and circulating blood volume [6, 7]. These antihypertensive drugs are extensively used for the treatment of hypertension and related cardiovascular diseases, but they are reported to have adverse side effects as well [5, 6]. According to Hom et al. [8], ACE inhibitor, angiotensin receptor blockers, and calcium channel blockers cause upper respiratory track abstraction and angioedema in adults and children. Calcium channel blockers contribute to the development and progression of cancer through the inhibition of vascular cell growth and angiogenic growth factors induced by the increase of apoptosis [9]. Other side effects of antihypertensive drugs, including dyspnea, cough, hair loss, headache, edema, and flushes, have been reported as well [10, 11]. Thus, an alternative therapy such as herbal drugs is preferred because natural herbal products using medicinal plants are considered to have fewer side effects [5]. Recently, medicinal plants have been reported to be effective in hypertension and empirically used as antihypertensive agents [12, 13]. The antihypertensive effects of plants are attributed to their antioxidant properties because oxidative stress is considered a risk factor in hypertension and cardiovascular diseases [14, 15]. Oxidant stress is caused by the imbalance between the generation of free radicals such as reactive oxygen and nitrogen species and antioxidant defense mechanisms [16]. Reactive oxygen species (ROS) produced in all vascular cells including endothelial, smooth muscle cells, and phagocytic cells play an important role in the pathophysiology of hypertension by causing vascular damage and reducing the production of nitric oxide (NO), which maintains the vascular tone [16, 17]. As such, excessive ROS is observed in patients with essential hypertension [18], with a close relationship between blood pressure and some parameters associated with oxidative stress reported [19]. Thus, antioxidants can promote the reduction of high blood pressure by trapping free radicals [16].

**Figure 1:**
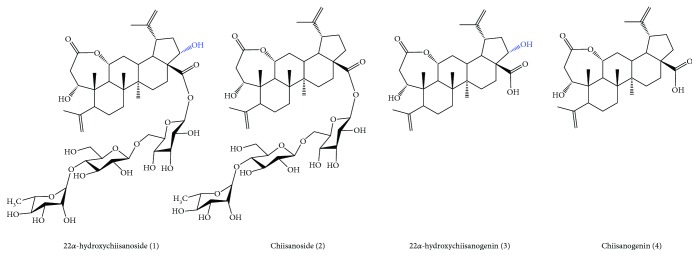
Structure of active compounds of *A. sessiliflorus* fruits.

*Acanthopanax sessiliflorus* (Rupr. & Maxim.) Seem., which belongs to the Araliaceae family, is reported to contain antioxidants [20]. It is widely found in the Far Eastern region of Russia and Northeast Asian countries including Korea, Japan, and China, and its stem and root are traditionally used for the treatment of rheumatoid arthritis, inflammation, and diabetes in oriental medicine [21–23]. In China, its fruits are used to develop various food therapy products because they are proven to be effective in cardiovascular and cerebrovascular diseases without toxicity. As such, Yang et al. [24] reported that the ethanolic extract of *A. sessiliflorus* fruits (DHP1501) had significant antithrombotic and antiplatelet activities. The activation of platelets occurs in hypertension, and platelet aggregation is involved in the development of vascular complications related to hypertension [25–27]. Interestingly, *A. sessiliflorus* fruits contain a high amount of secotriterpenoid glycosides, which are a member of the triterpenoid family, in addition to lignans and phenolics. Among these, chiisanoside, 22*α*-hydrochiisanoside, and their aglycone (Figure 1) are the major compounds of *A. sessiliflorus* fruits, showing effects on the anti-inflammation, antioxidant, and ACE inhibition using HUVECs (human umbilical vein endothelial cells) [21, 28–31]. Based on these results, we hypothesized that the extract of *A. sessiliflorus* fruits has potential antihypertensive effects. Therefore, we investigated our hypothesis in this study.

## 2. Materials and Methods

### 2.1. Preparation of Ethanolic Extract of *A. sessiliflorus* Fruits

**Figure 2:**
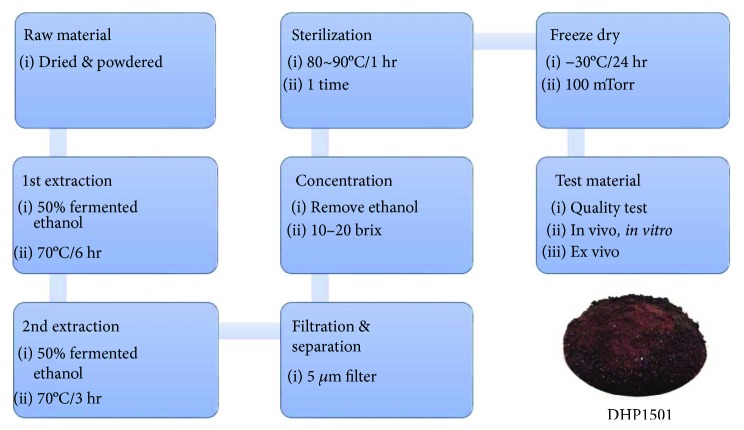
Manufacturing process for the production of DHP1501 powder.

*A. sessiliflorus* fruits were cultivated in Jeongseon, Gangwon Province, South Korea. A voucher specimen (NIHHS1501) was deposited at the Herbarium of the Department of Herbal Crop Research, National Institute of Horticultural and Herbal Science, Rural Development Administration, Eumseong, South Korea. The fruits of *A. sessiliflorus* were extracted with 50% aqueous fermented ethanol under reflux (70°C) for 6 h and extracted for a second time with 50% aqueous fermented ethanol under reflux (70°C) for 3 h. The extract was then filtered through a 5 *μ*m filter. The supernatant was vacuum-concentrated under reduced pressure to attain 10–20 brix materials. Then, it was sterilized at 80–90°C for 1 h. Finally, the ethanolic extract of *A. sessiliflorus* fruits (referred to as DHP1501) was freeze-dried by freeze dryer under reduced pressure (*−*30°C, 100 mTorr) for 24 h. To establish the bulk-scale production of DHP1501, we optimized the manufacturing process based on experiment scale (Figure 2).

### 2.2. Preparation of Sample and Standard Solutions for UPLC-QTOF/MS

Standard stock solutions of 22*α*-hydrochiisanoside (1), chiisanoside (2), 22*α*-hydrochiisanogenin (3), and chiisanogenin (4) were prepared by dissolving 1.00 mg each in 1 mL 70% methanol to yield a concentration of 1.00 mg/mL and were kept at 4°C. The standard stock solutions (1–4) were diluted with methanol to obtain calibration solutions with ranges of 0.5–10, 1–10, 0.5–15, and 0.5–20 mg/mL, respectively. 1.00 g of DHP1501 was accurately weighed and dissolved in fixed volumes (10 mL) of methanol, filtered through a 0.20 mm filter paper, and refrigerated at 4°C.

### 2.3. Analysis of Active Compounds Using UPLC-QTOF/MS

UPLC was performed using a Waters ACQUITY H-Class UPLC (Waters Corp.) with an ACQUITY BEH C18 column (2.1 *×* 100 mm, 1.7 *μ*m). The mobile phases consisted of water (A) with 0.1% formic acid (*v*/*v*) and acetonitrile (B) with 0.1% formic acid (*v*/*v*). The elution gradient was as follows: 0–4 min, B 10–30%; 4–15 min, B 30–60%; 15-16 min, B 60–100%; and 16–19 min, B 100–10%. The flow rate was 0.45 mL/min, and the injection volume was 2 *μ*l for each run. Next, HR-MS analysis was performed using Waters Xevo G2-S QTOF MS (Waters Corp.) operating in negative ion mode. The mass spectrometers performed alternating high- and low-energy scans known as MS^E^ acquisition mode. Accurate mass measurements were obtained by means of an automated calibration delivery system containing Leucine enkephalin, *m/z* 554.262 (ESI neg. mode), as internal reference. Optimal operating parameters were set as shown in Table 1 [32].

### 2.4. In Vitro Study

#### 2.4.1. Cell Culture

Human umbilical vein endothelial cells (HUVECs) were purchased from American Type Culture Collection (ATCC) and cultured at the Roswell Park Memorial Institute (RPMI) in 1640 medium (HyClone; GE Healthcare Life Sciences, UT, USA) with 10% FBS (HyClone; GE Healthcare Life Sciences) in humidified incubator with 5% CO_2_ and 37°C.

#### 2.4.2. Cell Viability Assay

Various concentrations (from 0.2 to 1000 *μ*g/mL) of DHP1501 were added to HUVECs seeded in a 96-well plate at a density of 5.0 × 10^4^ cells/mL and then incubated in a humidified incubator with 5% CO_2_ and 37°C for 24 h. Cell viability was measured by performing 3-(4,5-dimethylthiazol-2-yl)-2,5-diphenyltetrazolium bromide (MTT, Sigma-Aldrich, St. Louis, MO, USA) assay. In brief, 20 *μ*L of 5 mg/mL MTT was added, and further incubation was done for 4 h. The supernatant of each well was then replaced with 200 *μ*L of dimethylsulfoxide (DMSO, Sigma-Aldrich) to dissolve the formed formazan crystals. The absorbance (Ab) was determined at 595 nm with microplate reader (Spark 10M, TECAN, Männedorf, Switzerland). 0.5% DMSO was used as a vehicle control with 100% viability, and percent cell survival was calculated as follows: % of cell survival = [(Ab of treated group − Ab of blank group)/(Ab of vehicle control − Ab of blank group)] × 100.

#### 2.4.3. Intracellular NO Measurement

The production of NO in HUVECs was measured using diaminofluorescein-FM diacetate (DAF-FM/DA, Sigma-Aldrich) assay. Briefly, the cells seeded in a 96-well black plate at a density of 2 × 10^4^ cells/well were pretreated with DHP1501 (2, 20, or 200 *μ*g/mL) for 90 min before incubation in 5 *μ*M DAF-FM/DA for 1 h at 37°C. 0.5% DMSO was used as a vehicle control, with the relative levels of intracellular NO determined from the fluorescence intensity of DAF-FM at excitation of 486 nm and emission of 520 nm using fluorescence microplate reader (Spark 10M, TECAN).

#### 2.4.4. Electron Passaging Ability Determinations

The electron passaging abilities of DHP1501 were measured using 2,2-diphenyl-1-picrylhydrazyl (DPPH), 2,2′-azino-di-(3-ethylbenzthiazoline sulfonic acid) (ABTS), and oxygen radical absorption capacity (ORAC) assays. The DPPH radical (DPPH•) scavenging capacity assay, a decolorization assay, measures the capacity of antioxidants to react directly with DPPH radicals—which are stable organic nitrogen-centered free radicals whose dark purple color disappears when reduced to nonradical form by antioxidants—by monitoring absorbance at 517 nm. 5 *μ*L of DHP1501 at final concentrations of 125, 250, 500, and 1000 *μ*g/mL was added to 95 *μ*L of 0.3 mM DPPH solution and then incubated in the dark at 37°C. The negative control was prepared with the solvent used to dissolve DHP1501. The absorbance (Ab) was determined at 517 nm with microplate reader (Spark 10M, TECAN), and the radical scavenging activity was calculated as a percentage using the following equation: DPPH radical scavenging activity (%) = [1 − (Ab of treated group/Ab of negative control)] × 100. The total antioxidant capacity was determined using the colorimetric 6-hydroxy-2,5,7,8-tetramethylchroman-2-carboxylic acid (Trolox)-equivalent antioxidant capacity assay kit (Cayman Chemical, Ann Arbor, USA) according to the manufacturer's protocol. This assay is based on the ability of DHP1501 to inhibit the oxidation of ABTS® when incubated with peroxidase (metmyoglobin) and hydrogen peroxide [33]. The negative control was prepared with antioxidant assay buffer, solvent of DHP1501, instead of DHP1501 in the same manner, and color development was measured using microplate reader at 750 nm. Radical scavenging activity was calculated as a percentage using the following equation: ABTS radical scavenging activity (%) = [1 − (Ab of treated group/Ab of negative control)] × 100. The ORAC assay is based on the scavenging of peroxyl radicals generated by AAPH (2,2′-axobis-2-methyl-propanimidamide, dihydrochloride), which prevents the degradation of the fluorescein probe. ORAC antioxidant assay (Zen-Bio, Research Triangle Park, NC, USA) was performed based on the manufacturer's instructions. The reaction was performed in the wells of a microtiter plate containing DHP1501 (0.01, 0.1, or 1 mg/mL) to be tested and Trolox standard. Fluorescence was kinetically recorded every minute for 30 min using fluorescence microplate reader (excitation 485 nm; emission 530 nm). Areas under the fluorescence decay curve (AUC) were calculated using the following equation: AUC = 0.5 + (f1/f0) + (f2/f0) + ··· + 0.5 × (f31/f0) (f0 is normalized fluorescence at 0 min). The concentration of antioxidant in DHP1501 in proportion to the fluorescence intensity was assessed by comparing the net AUC to that of Trolox, a water-soluble vitamin E analog used as calibration standard (6.25, 12.5, 25, 50, and 100 *μ*M). The net AUC is determined by subtracting the AUC for no compound addition from the other AUC values. The results were expressed as *μ*M Trolox equivalents (TE) by the Trolox calibration standard.

#### 2.4.5. Antioxidant Activity Determinations through ROS Measurement

The antioxidant properties of DHP1501 was measured by CellROX and DCF-DA assay. Generation of ROS was measured by using CellROX™ Deep Red Reagent (Thermo Fisher scientific) and cell-permeant 2′,7′-dichlorodihydrofluorescein diacetate (H_2_DCFDA) (Thermo Fisher scientific). HUVECs were seeded in a 96-well plate at a density of 5.0 × 10^4^ cells/mL and then incubated in a humidified incubator with 5% CO_2_ and 37°C for 48 h. Thereafter, HUVECs were incubated under conditions of serum starvation for 4 h. After cell starvation, for CellROX assay, HUVECs were treated with the various concentrations (from 1, 10, 50, 100, and 200 *μ*g/mL) of DHP1501 with 1 mM H_2_O_2_ and then incubated in a humidified incubator with 5% CO_2_ and 37°C for 2 h. The cells were treated with CellROX Deep Red Reagent for 30 min at 37°C following the manufacturer's references. After washing three times with PBS, the fluorescence was obtained by using fluorescence microplate reader (ex./em. 640/665, Spark 10M, TECAN). For DCF-DA assay, HUVECs were treated with the various concentrations (from 10, 50, 100, and 200 *μ*g/mL) of DHP1501 with 500 *μ*M H_2_O_2_ and then incubated in a humidified incubator with 5% CO_2_ and 37°C for 3 h. Thereafter, the cells were treated with 25 *μ*M DCF-DA for 30 min at room temperature. After washing three times with HBSS, the fluorescence was detected by using fluorescence microplate reader (ex./em. 485/535, Spark 10M, TECAN).

### 2.5. Ex Vivo Study

#### 2.5.1. Preparation of Porcine Coronary Artery Rings

Porcine heart was obtained from the MK micropig (Scrofa domestica) provided by MEDIKINETICS (Gyeonggi-Do, Korea) and immediately immersed in cold normal saline. The coronary artery was dissected free from the surrounding myocardium and cleaned of any adherent fat and connective tissue. The artery was cut into rings with a diameter of 3 mm. The rings were suspended horizontally between two parallel stainless steel hooks for the measurement of isometric tension in organ bath containing Krebs solution (NaCl 118 mM, KCl 4.7 mM, MgSO_4_ 1.1 mM, KH_2_PO_4_ 1.2 mM, CaCl_2_ 1.5 mM, NaHCO_3_ 25 mM, and glucose 10 mM) and bubbled with a mixture of 95% O_2_ and 5% CO_2_. The temperature was maintained at 37°C throughout the experiment. The isometric tension generated by the coronary artery was measured using a force-displacement transducer (Hugo Sachs, Germany) and recorded with grass physiography (Hugo Sachs). In some rings, the endothelium was removed deliberately by rubbing the luminal surface gently with a wet cotton swab, and the absence or presence of endothelial cells was confirmed by the absence or presence of relaxation to the endothelium-dependent vasodilator bradykinin (300 nM) at 10 min after the rings were progressively contracted with concentrations of thromboxane A2 mimetic U46619 (from 1 to 60 nM) up to 80% of the maximum contraction.

#### 2.5.2. Relaxing Effect of DHP1501 on Precontracted Coronary Artery Rings

Coronary artery rings (*n* = 5) were contracted with U46619, and various concentrations of DHP1501 (from 0.001 to 1 mg/mL) were added when the U46619-induced contraction stabilized. *Ginkgo biloba* leaf extract (from 0.01 to 1 mg/mL) and 0.1% DMSO were used as positive control and negative control, respectively. Dose response curves in intact and endothelium-free rings were constructed, and concentrations of DHP1501 responsible for 50% of the relaxation (EC_50_) were determined.

### 2.6. In Vivo Study

#### 2.6.1. Animals

A total of 50 male spontaneously hypertensive rats (SHRs) and 10 Wistar-Kyoto rats (WKY) (aged 6 weeks) each weighing 180 ± 20 g were procured from a commercial breeder (Saeronbio, Inc., Gyeonggi-Do, Korea). We obtained institutional review board (IRB) approval for this study from the Korea Animal Medical Science Institute (KAMSI) (15-KE-216). The rats were housed under controlled environmental conditions (12 h light/dark cycle, temperature of approximately 23 ± 3°C, and humidity of 55 ± 15%) with food and water made available ad libitum throughout the experiments. Male Wistar-Kyoto rats were used as normal control (WKY), and SHRs were randomly divided into five groups (*n* = 10 in each group) according to body weight: (1) SHR (saline as the vehicle); (2) SHR-captopril 100 (a 4-week daily course of oral captopril at a dose of 100 mg/kg; (3) SHR-DHP1501 200 (a 4-week daily course of DHP1501 at a dose of 200 mg/kg, p.o.); (4) SHR-DHP1501 400 (a 4-week daily course of DHP1501 at a dose of 400 mg/kg, p.o.); and (5) SHR-DHP1501 600 (a 4-week daily course of DHP1501 at a dose of 600 mg/kg, p.o.).

#### 2.6.2. Blood Pressure Measurement

Systolic and diastolic blood pressures (SBP and DBP) were measured using noninvasive and invasive methods before and after oral administration of DHP1501. They were measured once weekly using a computerized tail-cuff plethysmograph (BP-2000; Visitech Systems, Apex, NC, USA), the noninvasive blood pressure measurement method. At the end of the experimental period, they were measured using MP36 (Biopac Systems Inc., USA), the invasive blood pressure measurement method, after carotid artery cannulation with PE20 tubes. Six measurements were obtained and averaged for each rats.

#### 2.6.3. Concentrations of Renin and Angiotensin I-Converting Enzyme (ACE)

The rats were anesthetized using pentobarbital sodium, and the blood samples were collected in a vacutainer tube containing clot activator from the inferior vena cava. The samples were kept at room temperature for 15*–*20 min and centrifuged at 3000 rpm for 10 min to obtain the serum. The serum was aliquoted and stored in an ultra-low temperature freezer (−70°C) for the analysis of ACE concentration. Concentrations of ACE and renin were determined by ELISA kits based on the manufacturer's instructions (Rat REN (Renin) ELISA kit, Elabscience Biotechnology Co. Ltd., WuHan, China; Rat ACE ELISA kit, Elabscience Biotechnology) and expressed in ng/mL.

### 2.7. Statistical Analysis

The statistical analysis was conducted with one-way analysis of variance (ANOVA) and Kruskal-Wallis test using the Prism 5.03 (GraphPad Software Inc., San Diego, CA, USA). One-way analysis of variance (ANOVA) was followed by the Student–Newman–Keuls test for multiple comparisons. The significance level for all analyses was set a priori at *p* ≤ 0.05, and all values were expressed as the mean ± standard deviation (SD).

## 3. Results

### 3.1. Identification of Standard Compounds in DHP1501

**Figure 3:**
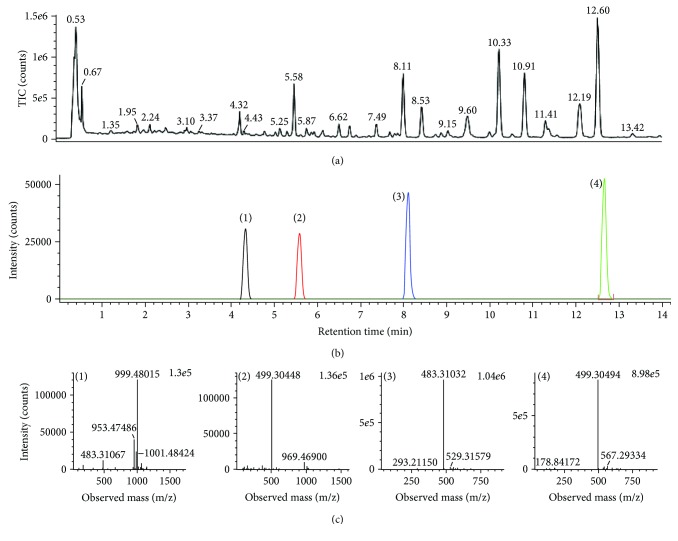
TIC of DHP1501 (a), four standard analytes (b) by UPLC-QTOF/MS in a negative ion mode by selected ion monitoring, and representative QTOF/MS chromatograms (c) for 22*α*-hydrochiisanoside (1), chiisanoside (2), 22*α*-hydrochiisanogenin (3), and chiisanogenin (4).

Standard compounds (1*–*4) were isolated and purified from the fruits of *A. sessiliflorus* by a series of chromatography procedures in our laboratory, and their structures were elucidated by a comparison of spectroscopic data (MS, ^1^H-NMR, and ^13^C-NMR) with the literature data: 22*α*-hydrochiisanoside, chiisanoside, 22*α*-hydrochiisanogenin, and chiisanogenin [34, 35]. The purity of the isolated compounds was determined to be more than 99% by the normalization of the peak areas detected by UPLC analysis. Since UPLC-QTOF/MS has been proven to be a suitable tool for the identification of the four compounds, the separation of constituents in DHP1501 was performed by UPLC-QTOF/MS in a negative ion mode.  Figure 3 shows a typical total ion chromatogram (TIC) of the identified compounds with mass detection.

### 3.2. Quantitative Analysis of DHP1501 by UPLC-QTOF/MS

Linear calibration curves were obtained for four compounds at different concentration levels. The characteristics of the calibration plots are summarized in Table 2. As seen in the table, the four compounds show excellent correlation coefficients. Detector counts (relative peak area) were linearly dependent on sample concentration over the range of 0.5–1 *μ*g/mL for 1, 1–10 *μ*g/mL for 2, 0.5–15 mg/mL for 3, and 0.5–20 mg/mL for 4. The LODs of 22*α*-hydrochiisanoside, chiisanoside, 22*α*-hydrochiisanogenin, and chiisanogenin were 0.002, 0.002, 0.003, and 0.009 ppm, respectively. The LOQs of 22*α*-hydrochiisanoside, chiisanoside, 22*α*-hydrochiisanogenin, and chiisanogenin were determined to be 0.006, 0.006, 0.009, and 0.009 ppm, respectively, by UPLC-QTOF/MS in a negative ion mode. The amount of 22*α*-hydrochiisanoside, chiisanoside, 22*α*-hydrochiisanogenin, and chiisanogenin in the DHP1501 obtained using validation methods (Table 2) was 0.69, 1.47, 1.65, and 2.71 mg/g, respectively.

### 3.3. Evaluation of Cell Cytotoxicity of DHP1501

**Figure 4:**
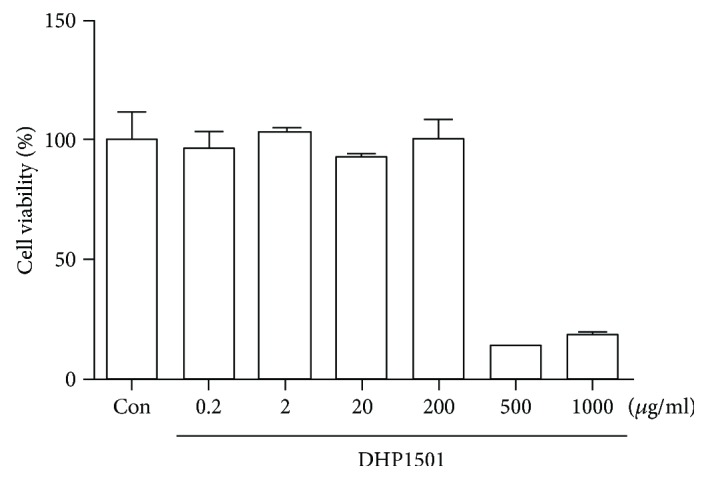
Cell viability of DHP1501. Cell viability was expressed as percentage versus vehicle control (100%), and values were expressed as the mean ± standard deviation (SD).

The *in vitro* cytotoxicity of DHP1501 was determined in HUVECs using MTT assay. Cell viability was 96.70 ± 6.30, 103.20 ± 1.55, 93.08 ± 1.06, 100.22 ± 7.95, 13.85 ± 0.36, or 18.69 ± 0.49% at concentration of 0.2, 2, 20, 200, 500, or 1000 *μ*g/mL, respectively (Figure 4). DHP1501 at concentrations ranging from 0.2 to 200 *μ*g/mL did not exhibit toxicity on HUVECs, but 500 or 1000 *μ*g/mL of DHP1501 showed cytotoxicity.

### 3.4. Effects of DHP1501 on NO Production in HUVECs

**Figure 5:**
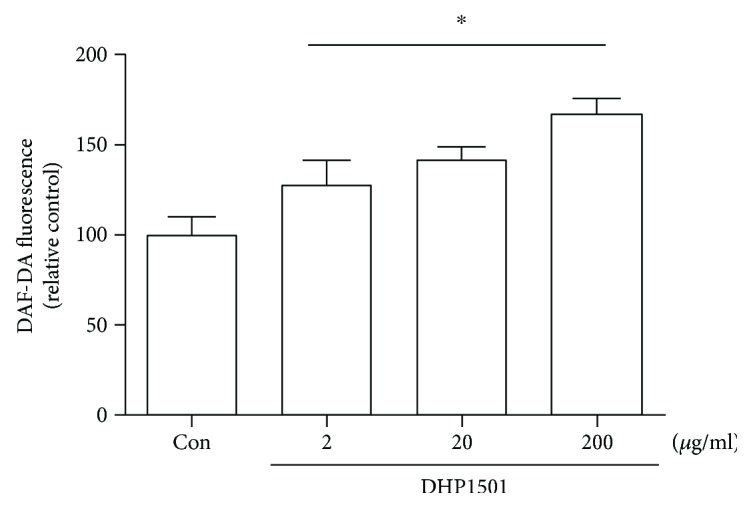
NO production effects of DHP1501 on HUVECs. NO generation was measured by using DAF-DA assay. HUVECs were treated with DHP1501 at concentrations ranging from 0.2 *μ*g/mL to 200 *μ*g/mL, or the same volume of vehicle (Con, 0.5% DMSO in distilled water). Data represent the mean ± SD (*n* = 6/group) (^∗^*p* < 0.05, versus the vehicle-treated controls).

We investigated whether DHP1501 affects endothelial NO generation. When the DAF-FM fluorescence intensity of vehicle control was set as 100%, the fluorescence intensity was 127.54 ± 14.10, 141.47 ± 8.16, or 167.54 ± 8.41% in 2, 20, or 200 *μ*g/mL, respectively (Figure 5). DHP1501 at the tested concentrations facilitated the endothelial NO production in a dose-dependent, significant manner (F_3, 23_ = 43.94, *p* < 0.05).

### 3.5. Electron Passaging Abilities of DHP1501

**Figure 6:**
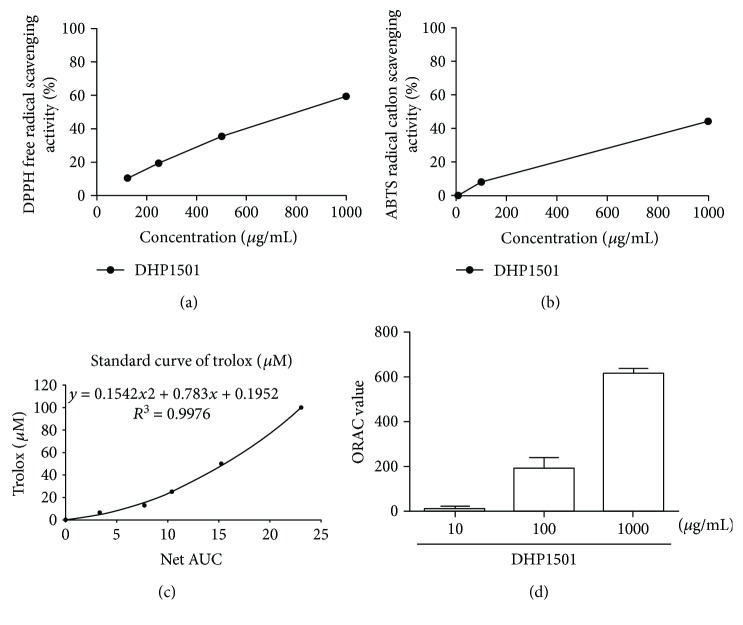
The electron passaging properties of DHP1501. (a) DPPH radical scavenging activity. (b) ABTS radical scavenging activity measured by ORAC assay. (c) Trolox standard curve (or decay curve of fluorescence). (d) ORAC values expressed as TE.

The electron passaging properties of DHP1501 were determined by DPPH, ABTS, and ORAC assays. As shown in Figure 6, DHP1501 exhibited the scavenging activity of DPPH, ABTS, and peroxyl radicals. The DPPH radical scavenging activity of DHP1501 was 10.31 ± 2.41, 19.27 ± 0.95, 35.35 ± 0.80, or 59.22 ± 2.17 in 125, 250, 500, or 1000 *μ*g/mL, respectively (Figure 6(a)). The ABTS radical scavenging activity of DHP1501 was 0.08 ± 0.17, 8.32 ± 1.87, or 43.88 ± 2.93% in 10, 100, or 1000 *μ*g/mL, respectively (Figure 6(b)). When the peroxyl radical scavenging activity was expressed as *μ*M Trolox equivalents (TE), DHP1501 was 11.36 ± 10.89, 190.44 ± 47.36, or 614.02 ± 22.82 *μ*M TE in 10, 100, or 1000 *μ*g/mL, respectively (Figures 6(c) and 6(d)).

### 3.6. Antioxdant Activity of DHP1501 through ROS Measurement

**Figure 7:**
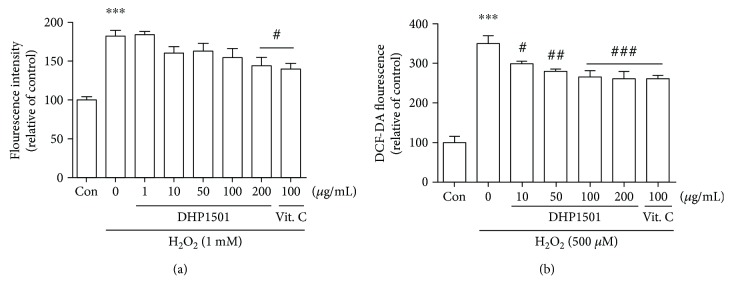
Antioxidant activity of DHP1501 through ROS measurement. (a) Fluorescence signal intensity (CellROX). (b) Fluorescence signal intensity (DCF-DA). Values were expressed as mean ± SD (*n* = 5 − 6/group) (^∗∗∗^*p* < 0.05, versus the vehicle-treated control group; ^#/##/###^*p* < 0.05/*p* < 0.01/*p* < 0.001, versus the vehicle-treated H_2_O_2_ group).

The antioxidant properties of DHP1501 were determined by CellROX and MitoSOX assays. As shown in Figure 7(a), in CellROX assay, an increase of fluorescence intensity was observed in the H_2_O_2_-treated group (181.3 ± 19.17), which was significantly reversed by the treatment of 200 *μ*g/mL of DHP1501 (143.8 ± 23.56) and Vitamin C (139.6 ± 16.37), as positive control (F_7, 32_ = 9.88, *p* < 0.05). As shown in Figure 7(b) in DCF-DA assay, an increase of fluorescence intensity was observed in the H_2_O_2_-treated group (350.1 ± 46.60), which was significantly reversed by the treatment of DHP1501 (261.6 ± 42.46) and Vitamin C (261.7 ± 18.39) (F_6, 35_ = 31.88, *p* < 0.05). It shows that the treatment of DHP1501 suppressed the H_2_O_2_-induced accumulation of ROS in HUVECs.

### 3.7. Effects of DHP1501 on Vasorelaxation

**Figure 8:**
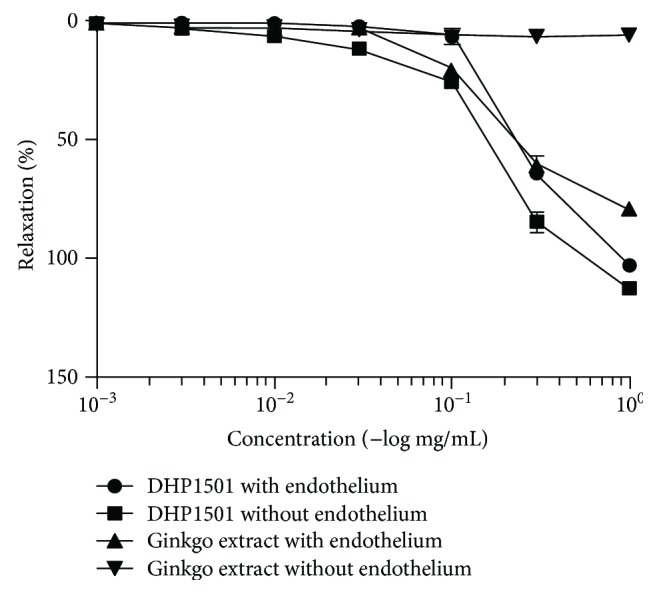
Effects of DHP1501 on relaxation in porcine coronary artery rings. Rings were contracted with U46619. Values were expressed as the mean ± SD in terms of percentage relaxation of the contraction to U46619 (*n* = 5/group). Relaxation induced by DHP1501 in intact rings or endothelium free rings. Values were expressed as mean ± SD.

DHP1501 was determined for the potency of concentration-dependent relaxations of porcine coronary artery rings (Figure 8). The EC_50_ value of the vasorelaxing effect on endothelium-intact coronary artery rings was 0.26 ± 0.03 mg/mL or 0.29 ± 0.06 mg/mL in DHP1501 or *Ginkgo biloba* leaf extract as the positive control, respectively. The EC_50_ value for the endothelium-free coronary artery rings was 0.16 ± 0.03 mg/mL in DHP1501, but no vasorelaxing effect by ginkgo extract was observed. Specifically, DHP1501 reduced contractile responses to thromboxane mimetic U46619 in porcine coronary rings in a dose-dependent manner regardless of endothelium.

### 3.8. Effects of DHP1501 on Blood Pressure in SHRs

**Figure 9:**
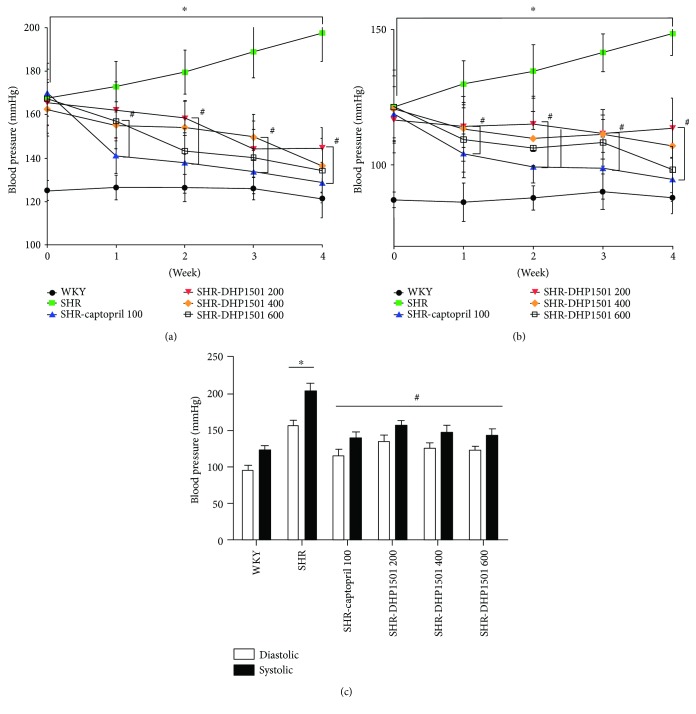
Effects of DHP1501 on blood pressure; SBP and DBP. (a) Changes in SBP and DBP induced by DHP1501 for 4 weeks. (b) Changes in SBP and DBP induced by DHP1501 at the end of the experiment period. Groups included the following: WKYs as normal control; SHR (saline as the vehicle); SHR-captopril 100 (a 4-week daily course of oral captopril at a dose of 100 mg/kg); SHR-DHP1501 200 (a 4-week daily course of oral DHP1501 at a dose of 200 mg/kg); SHR-DHP1501 400 (a 4-week daily course of oral DHP1501 at a dose of 400 mg/kg); and SHR-DHP1501 600 (a 4-week daily course of oral DHP1501 at a dose of 600 mg/kg). (c) Effect of DHP1501 on SBP and DBP measured by an invasive method. Values were expressed as the mean ± SD (*n* = 10/group) (^∗^*p* < 0.05, versus the WKY group; ^#^*p* < 0.05, versus the SHR group).

At the beginning of the experiment, SBP and DBP were significantly higher in the SHR groups than in the WKY. SBP and DBP in SHR were gradually increased during the experiment period and were the highest at 4 weeks (197.5 ± 13.0 and 148.5 ± 8.0 mmHg in SBP and DBP, resp.). On the other hand, SBP and DBP in SHR-captopril as positive control were significantly reduced after 1 week of treatment and were maintained at the lowest levels during the experiment period compared with SHR (SBP: F_5, 54_ = 26.68; DBP: F_5, 54_ = 21.42). Oral administration of DHP1501 reduced SBP and DBP in a dose-dependent manner at 1 week and significantly decreased them after 2 weeks compared with SHR. Especially, 400 or 600 mg/kg of DHP1501 had no significant difference with SBP and DBP compared to the SHR-captopril group at 4 weeks (Figures 9(a) and 9(b)).

Similarly, SBP and DBP measured by an invasive method at the end of the experiment were significantly higher in the SHR compared to the WKY (121.3 ± 7.4 versus 202.2 ± 11.2 mmHg in SBP and 94.9 ± 6.5 versus 155.4 ± 7.6 mmHg in DBP). SBP and DBP were significantly decreased in SHR-captopril compared with SHR (137.9 ± 9.3 and 114.3 ± 9.1 mmHg, respectively) and reduced in a dose-dependent manner in SHR-DHP1501 200 (154.9 ± 8.6 and 133.2 ± 9.4 mmHg), SHR-DHP1501 400 (146.1 ± 10.2 and 124.7 ± 7.7 mmHg), and SHR-DHP1501 600 (142.1 ± 9.6 and 121.4 ± 6.3 mmHg). The decrease in SBP and DBP as induced by DHP1501 was statistically significant compared with the SHR (SBP: F_5, 54_ = 84.72; DBP: F_5, 54_ = 65.21) (Figure 9(c)).

### 3.9. Effects of DHP1501 on Renin and ACE Inhibitory Activity

**Figure 10:**
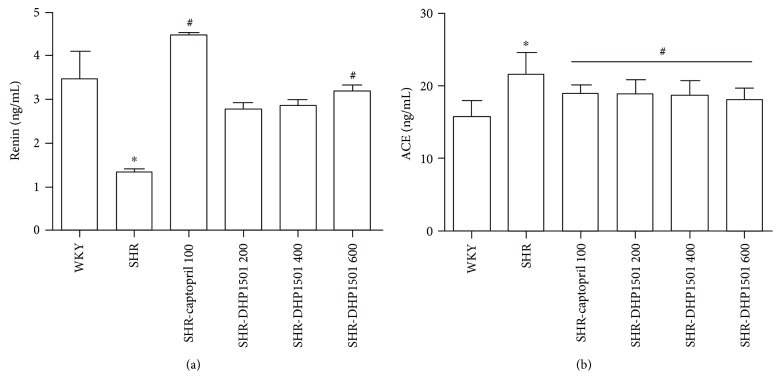
Effects of DHP1501 on serum renin (a) and ACE concentrations (b). (a) Changes in renin concentration induced by DHP1501. (b) Changes in ACE concentration induced by DHP1501. Groups included the following: WKYs as normal control; SHR (saline as the vehicle); SHR-captopril 100 (a 4-week daily course of oral captopril at a dose of 100 mg/kg); SHR-DHP1501 200 (a 4-week daily course of oral DHP1501 at a dose of 200 mg/kg); SHR-DHP1501 400 (a 4-week daily course of oral DHP1501 at a dose of 400 mg/kg); and SHR-DHP1501 600 (a 4-week daily course of oral DHP1501 at a dose of 600 mg/kg). Values were expressed as the mean ± SD (*n* = 10/group) (^∗^*p* < 0.05, versus the WKY group; ^#^*p* < 0.05, versus the SHR group).

The serum renin concentration was remarkably decreased in SHR compared with WKY (1.34 ± 0.18 versus 3.48 ± 0.63 ng/mL, resp.). Note, however, that the DHP1501-treated group had significantly lower serum renin concentration compared with the SHR group. The SHR-captopril group recorded a conspicuous increase (4.46 ± 0.21 ng/mL). Similar to captopril used as a positive control, DHP1501 increased the serum renin concentrations—2.78 ± 0.42, 2.85 ± 0.47, or 3.19 ± 0.41—in SHR-DHP1501 200, SHR-DHP1501 400, or SHR-DHP1501 600, respectively, in a dose-dependent manner (Figure 10(a)). Contrary to serum renin, the serum ACE concentration was significantly increased in SHR compared with WKY (21.57 ± 3.03 versus 15.81 ± 2.12 ng/mL, resp.). On the other hand, in terms of serum ACE concentration, significant group effects were observed in the DHP1501-administered group (F_5,54_ = 7.616). Specifically, serum ACE concentration was 18.92 ± 1.26, 18.76 ± 2.12, 18.66 ± 2.10, or 18.05 ± 1.63 ng/mL in SHR-captopril, SHR-DHP1501 200, SHR-DHP1501 400, or SHR-DHP1501 600, respectively (Figure 10(b)).

## 4. Discussion

The endothelium forming the interior surface of blood vessels is involved in the regulation of vascular tone by releasing several mediators and maintaining balance between vasoconstriction and vasorelaxation [36]. Especially, the NO generated by endothelial nitric oxide synthase (eNOS) in endothelial cells mediates vascular relaxation and controls cardiovascular homeostasis [37, 38]. In the vascular system, NO binds to Fe^2+^ heme of soluble guanylyl cyclase (sGC) and activates sGC, resulting in the production of cyclic guanosine-3′,5′-monophosphate (cGMP). As the second messenger, cGMP activates protein kinase G as well as the signaling cascade, such as K^+^ channels. The activation of K^+^ channels hyperpolarizes the cell membrane and blocks Ca^2+^ channels, leading to vascular relaxation [39].

ROSs including superoxide (O_2_-) react with the NO produced by eNOS and yield unstable molecules such as peroxynitrite (ONOO-) and peroxynitrous acid (HNO3), impairing endothelium-mediated vasorelaxation [40, 41]. Thus, antioxidants are reported to be beneficial in preventing endothelial dysfunction by scavenging superoxide and peroxynitrite [42]. *Acanthopanax sessiliflorus* fruit, which is colored berry, contains phytochemicals such as anthocyanin, total polyphenol, and total flavonoid, contributing to the antioxidant activity with health protection ability [43]. Many medicinal plants also reportedly contain a lot of antioxidants such as polyphenol; thus, the antioxidant activity of the extracts from natural resources is closely correlated with their total phenolic compounds possessing one or more aromatic rings with one or more hydroxyl groups [43–45]. In this present study, DHP1501 showed the electron passaging properties by scavenging DPPH, ABTS, and peroxyl radicals (Figure 6) and showed biologically relevant antioxidant activity in CellROX and DCF-DA assays (Figure 7). Moreover, the dosages of DHP1501 which did not cause cytotoxicity in the vascular endothelial cells significantly facilitated the endothelial NO production (Figures 4 and 5). Decreased NO levels are associated with impaired endothelium-dependent vasodilation in many cardiovascular diseases, whereas increase in endothelial-dependent NO release is considered to be beneficial to the cardiovascular system by improving vasodilation and blood circulation [46, 47]. Vasorelaxation is correlated with hypertension because structural and functional changes in the vascular endothelium contribute to hypertension [48]. Our study showed that DHP1501 possessed strong potential to induce full relaxation in the contracted vessels. In the endothelial-intact porcine coronary rings, vasorelaxation induced by DHP1501 was similar to that of the *Ginkgo biloba* leaf extract, which is proven to possess definite dose-dependent antihypertensive activity [49]. Additionally, DHP1501 exhibited effective vasorelaxation in the endothelium-free rings unlike the *Ginkgo biloba* leaf extract (Figure 8).

The antihypertensive effects of DHP1501 were definitively confirmed in SHRs, which have been widely used to investigate the antihypertensive effects of natural products or foods [50]. Following oral administration of various concentrations of DHP1501 or captopril, SBP and DBP significantly decreased. Especially, a high dose of DHP1501 (600 mg/kg) decreased blood pressure to levels comparable to the captopril-administered group (Figures 9(a) and 9(b)).

The renin-angiotensin-aldosterone system (RAAS) plays a crucial role in regulating blood pressure in the body [51]. Generally, renin activates RAAS by cleaving angiotensinogen and yields angiotensin I, which is further converted into angiotensin II, a potent vasoconstrictor. Eventually, renin causes an increase in blood pressure. Thus, elevated renin levels in SHR, which is an established model of essential hypertension, have been reported in several studies [52–54]. Still, there is controversy on the renin level in SHR because other studies have reported normal or subnormal levels of renin in SHR compared with a normotensive Wistar rat [55, 56]. Sen et al. [57] also showed that plasma renin activity was significantly higher in SHR at an early age, decreasing with the development of stable hypertension and finally becoming lower than normal in established hypertension. They suggested that renin may play a primary role along with other possible factors in the pathology of hypertension. Recently, Qu et al. reported that the renin content in the SHR groups was lower than that in the age-matched WKY rats, and similar trends were found in the human plasma. They also stated that renin levels were negatively correlated with blood pressure [58]. In this study, too, serum renin concentration in SHR was conspicuously lower than that in WKY, although SBP and DBP were significantly higher in SHR than WKY. On the other hand, captopril and DHP1501 significantly increased serum renin concentration in SHR (Figure 9). According to Bolterman et al., captopril, an ACE inhibitor, effectively treated high blood pressure even as levels of plasma renin activity in the SHR are normal, thus suggesting that angiotensin II plays a major role in the etiology of hypertension in SHR. Finally, they concluded that captopril may decrease blood pressure in SHR by selectively decreasing angiotensin II, oxidant stress, and endothelium involved in the increase of blood pressure [59]. Similar to captopril, DHP1501 reduced the serum ACE in SHRs (Figure 9(b)) in a dose-dependent, significant manner. Our previous study also reported that DHP1501 decreased ACE activity in HUVECs [31]. In RAAS, ACE converts angiotensin I into angiotensin II, which is considered a potent vasoconstrictor, and degrades bradykinin—a potent endothelium-dependent vasodilator—by releasing prostacyclin, NO, and endothelium-derived hyperpolarizing factor [60–62]. Therefore, the inhibition of ACE has been extensively used as a therapeutic strategy for the prevention and treatment of hypertension and has been found to improve endothelium-dependent vasorelaxation [6, 48]. As a follow-up study, we will evaluate the unexpected side effects of DHP1501 at WKY rats.

## 5. Conclusions

Overall, we investigated the antihypertensive effect of DHP1501, which may be attributed to various factors such as free radical scavenging capacity, facilitation of NO production, and ACE inhibition, resulting in the improvement of vascular relaxation and decrease in blood pressure in the hypertensive animal model. These results suggest that DHP1501 may be a promising functional material for the prevention and treatment of hypertension. The advantages of hybrid Q-TOF mass spectrometry include not only quality detection capability and sensitivity but also accurate measurement and reliable chemical fragmentation, making the structure elucidations easier. It can be used for qualitative and quantitative determination of active compounds, which is helpful in improving the quality control of ethanolic extract from *A. sessiliflorus* fruits and its pharmaceutical preparations.

## Figures and Tables

**Table 1:** Optimal conditions for the QTOF/MS analysis of DHP1501 powder^a^.

Optimal Q-TOF/MS condition	
Ion source	ESI negative mode
Source temp. & desolvation temp.	120°C/550°C
Cone gas flow & desolvation gas flow	30 L/h/800 L/h
Capillary volt & cone volt	3 k/40 V
Mass range (*m/z*)	50 to 1.000
Collision energy range	4 to 45 eV

^a^Table 1 is reproduced from Lee et al. [32].

**Table 2:** Linear regression data and contents of the validated method for the investigated compounds (1–4) in DHP1501^a^.

Compounds	Rt^b^ (min)	Calibration curve^c^	*R* ^2^	Line arrangement (*μ*g/mL)	LOD^d^ (ppm)	LOQ^e^ (ppm)	Amount (mg/g)
1	4.33	*y* = 765.07*x* + 150.61	0.995	0.5–10	0.002	0.006	0.69
2	5.59	*y* = 744.66*x* + 352.97	0.993	1–10	0.002	0.006	1.47
3	8.11	*y* = 2567*x* + 1764.10	0.995	0.5–15	0.003	0.009	1.65
4	12.66	*y* = 3119*x* + 2417.80	0.993	0.5–20	0.009	0.009	2.71

^a^Mean values of samples (*n* = 3). ^b^Rt: retention time. ^c^*y*: logarithmic value of peak area; *x*: logarithmic value of amount injected. ^d^LOD: limit of detection. ^e^LOQ: limit of quantification.

## Abbreviations

ACE: Angiotensin-converting enzyme
ABTS: 2,2′-Azino-di-(3-ethylbenzthiazoline sulfonic acid)
Ab: Absorbance
ANOVA: One-way analysis of variance
AUC: Areas under the fluorescence decay curve
cGMP: Cyclic guanosine-3′,5′-monophosphate
DAF-FM/DA: 4-Amino-5-methylamino-2′,7′-difluorofluorescein diacetate
DBP: Diastolic blood pressure
DHP1501: The ethanolic extract of *A. sesssilflorus*
DPPH: 2,2-diphenyl-1-picrylhydrazyl
eNOS: Endothelial nitric oxide synthase
HUVECs: Human umblical vein endothelial cells
IRB: Institutional review board
KAMSI: Korea Animal Medical Science Institute
LOD: Limit of detection
LOQ: Limit of quantification
NO: Nitric oxide
ORAC: Oxygen radical absorption capacity
RAAS: Renin-angiotensin-aldosterone system
REN: Renin
Rt: Retention time
SBP: Systolic blood pressure
SD: Standard deviation
sGC: Soluble guanylyl cyclase
SHRs: Spontaneous hypertensive rats
TE: Trolox equivalents
TIC: Total ion chromatogram
Trolox: 6-Hydroxy-2,5,7,8-tetramethylchroman-2-carboxylic acid
WKY: Wistar-Kyoto rats.
